# Advanced Vascular Access in Small Animal Emergency and Critical Care

**DOI:** 10.3389/fvets.2021.703595

**Published:** 2021-11-29

**Authors:** Jack A. Lee, Liz-Valéry S. Guieu, Geneviève Bussières, Christopher K. Smith

**Affiliations:** Small Animal Clinical Sciences, College of Veterinary Medicine, University of Tennessee, Knoxville, TN, United States

**Keywords:** difficult vascular access, intraosseous, cutdown, emergency, veterinary, ultrasound

## Abstract

In canine and feline patients presenting in a state of hemodynamic collapse, obtaining vascular access can be challenging. Delays in achieving vascular access interfere with delivery of patient care. In human medicine, definitions of difficult vascular access are variable and include the need for multiple placement attempts or involvement of specialized teams and equipment. Incidence and risk factors for difficult vascular access have not been well studied in veterinary patients, which limits understanding of how best to address this issue. Alternatives to percutaneous peripheral or central intravenous catheterization in dogs and cats include venous cutdowns, umbilical access in newborns, corpus cavernosum access in males, ultrasound-guided catheterization, and intraosseous catheterization. In recent years, advances in ultrasonography and intraosseous access techniques have made these more accessible to veterinary practitioners. These vascular access techniques are reviewed here, along with advantages, limitations, and areas for future study of each technique.

## Introduction

Multiple definitions of difficult vascular access (DVA) exist in the human medical literature, which encompass the need for multiple placement attempts, specialized equipment, and highly experienced teams (1–3). In human pediatrics, ~50% of patients are successfully catheterized on the first attempt; 5–33% of patients require more than two placement attempts; and in one study, 5% could not have a peripheral intravenous (IV) catheter placed at all (4, 5). Children below 2 years old are the most challenging and time-consuming to catheterize, which is thought to be due to their smaller size, difficulty in palpating vasculature, and possibly patient non-compliance (1, 3, 5). In pediatrics, scoring systems such as the difficult IV access (DIVA) scoring tool based on age, vein visualization, and vein palpation have been developed and may be helpful in identifying patients at high risk for DVA (5, 6). A similar scoring system has also been developed for adults based on vessel visibility, palpability, and size, as well as a history of DVA and emergency indication for surgery (7, 8). Besides age, factors that may contribute to DVA in humans include dehydration, hypotension, metabolic disease such as diabetes mellitus, cardiovascular disease, scarring from recurrent catheterization, skin lesions associated with trauma or burn injury, and obesity (2, 9–11). The consequences of DVA can be severe, including discomfort associated with repeated attempts and delays in necessary treatments, which can be life-threatening (12–14). In human medicine, use of dedicated vascular access teams for selection, placement, and maintenance of vascular access devices helps to improve success rates and decreases complications (2, 15). These teams combine dedicated nurses, technicians, and doctors with formal training; teams may subspecialize in specific procedures (e.g., peripheral catheters and central venous catheters). Recently, the development of smartphone-based applications has also been investigated to help with vein identification based on multispectral Wiener estimation using the phone camera (16, 17). These appear to be a helpful point-of-care way to improve vein visualization but have not been clinically investigated. Other phone applications have been developed to aid in appropriate vascular access device selection in individual patients (18). To the authors' knowledge, these have not been evaluated for use in veterinary patients.

A consensus definition of DVA does not exist in veterinary medicine, and its prevalence in canine and feline patients has not been well-reported, particularly in critically ill patients. Successful first-time peripheral catheter placement rates in dogs and cats have been documented in 51–94% of attempts in a general patient population (19, 20), with individual experience being an important factor for success (19). In the critically ill veterinary patient, the incidence of DVA can be expected to increase given the multitude of presenting comorbidities such as hypotension, vasoconstriction, trauma to the desired access sites, and non-compliance.

Gaining venous access is vital to patient care and allows for blood sampling and delivery of therapies such as medications, fluids, and blood products. The most common initial approach is typically peripheral IV catheterization, while central catheterization or other advanced vascular access options may be considered in certain patients. In general, short, small-diameter catheters decrease the risk of thrombophlebitis (21–23). Short, large-gauge catheters allow for more rapid administration of fluids (24). With longer catheters, rapid delivery of fluids can be challenging due to the proportional increase in resistance that accompanies length, as described by the Hagen–Poiseuille equation. This is particularly true in smaller-bore catheters (24, 25). A number of alternatives to traditional peripheral and central IV catheterization have become available to small animal practitioners in recent years and are described here.

## Venous Cutdown

In cases when visualization or palpation of the vessel is challenging, peripheral and central catheter placement can be facilitated with a cutdown. This can be achieved with readily available supplies, though depending on operator skill can be more time-consuming than some alternatives. The cephalic, lateral saphenous, or jugular veins are common sites for cutdown approaches in dogs. In dogs with large ears, such as Basset Hounds and Dachshunds, the auricular vein approach can also be used. In cats, the medial saphenous approach can also be used (26, 27). The technique is described elsewhere (26, 27). Contraindications include trauma to the desired placement area or anywhere along the limb proximal to the insertion site along the course of the desired vessel. Infection at the desired site is an additional contraindication. Coagulopathy is a relative contraindication, particularly for larger vessels where the risk of life-threatening hemorrhage should be considered and other techniques pursued if possible (28). Complications may include vessel damage, hemorrhage, hematoma, local infection, thrombosis, and nerve injury (26, 27). Cutdowns may have an increased risk of infection and hemorrhage compared with conventional catheter placement (20–23). The frequency of complications in humans has been reported to be between 2 and 15%, though recent data are lacking (28, 29). Complications are minimized by removal of the catheter as soon as another option is available (24, 29). The average time to perform the technique in humans has been reported to be between 5.6 and 7.5 min (30, 31). Successful peripheral IV catheter placement with a cutdown was reported as 69% in a cadaver study and 85% in a hypotensive population (29, 31). In humans, use of the Seldinger technique and other advanced vascular access options have made cutdowns less popular (28). Use of cutdowns in veterinary medicine has been sparsely documented. To the authors' knowledge, cutdowns have not been clinically studied in veterinary patients, nor has the rate of complications been reported. Further investigation is needed to compare outcomes with other available options in veterinary patients.

As an alternative to full cutdown, a technique described as facilitative incision or relief hole can be utilized for severely dehydrated patients, or those with thick skin, to reduce the tension and friction of the skin against the catheter (27). A skin incision of about 1–2 mm is made directly over the vessel extending through the dermis using a number 11 blade or appropriately sized hypodermic needle with or without local anesthesia (rarely needed). Care should be taken to avoid the vessel when making the relief incision. This technique, if successful, has less chance of infection or healing issues but does not allow for visualization of the vein (27).

## Corpus Cavernosum

An infrequently utilized alternative to peripheral venous catheterization for male patients with DVA is catheterization of the corpus cavernosum. This technique is effective due to the substantial venous drainage from this structure and requires no specialized equipment. After aseptic preparation, access is achieved using a 19-G catheter inserted at an oblique angle toward the radix through the skin and into the corpus cavernosum (located on the lateral aspect of the penis, caudal to the os penis) (32–34). Placement is confirmed *via* aspiration of blood. One known limitation would be in pelvic or penile trauma, where venous return may be impaired. Human literature on resuscitation using this technique is limited (35), though the treatment of erectile dysfunction using injections into the corpus cavernosum suggests that while the potential for complications such as fibrosis and penile dysfunction are possible, they are rare (34). In canine experimental models, placement of a catheter into the corpus cavernosum achieved sufficient flow rates to resuscitate experimentally induced hypovolemic animals, as well as to administer epinephrine and atropine (33, 36). Administration of blood products and phenobarbital has also been successful (32–34). Administration of other emergency medications has not been studied, and further clinical work is required to fully assess the incidence of complication and limitations of this modality.

## Ultrasound Guidance

**Figure 1:**
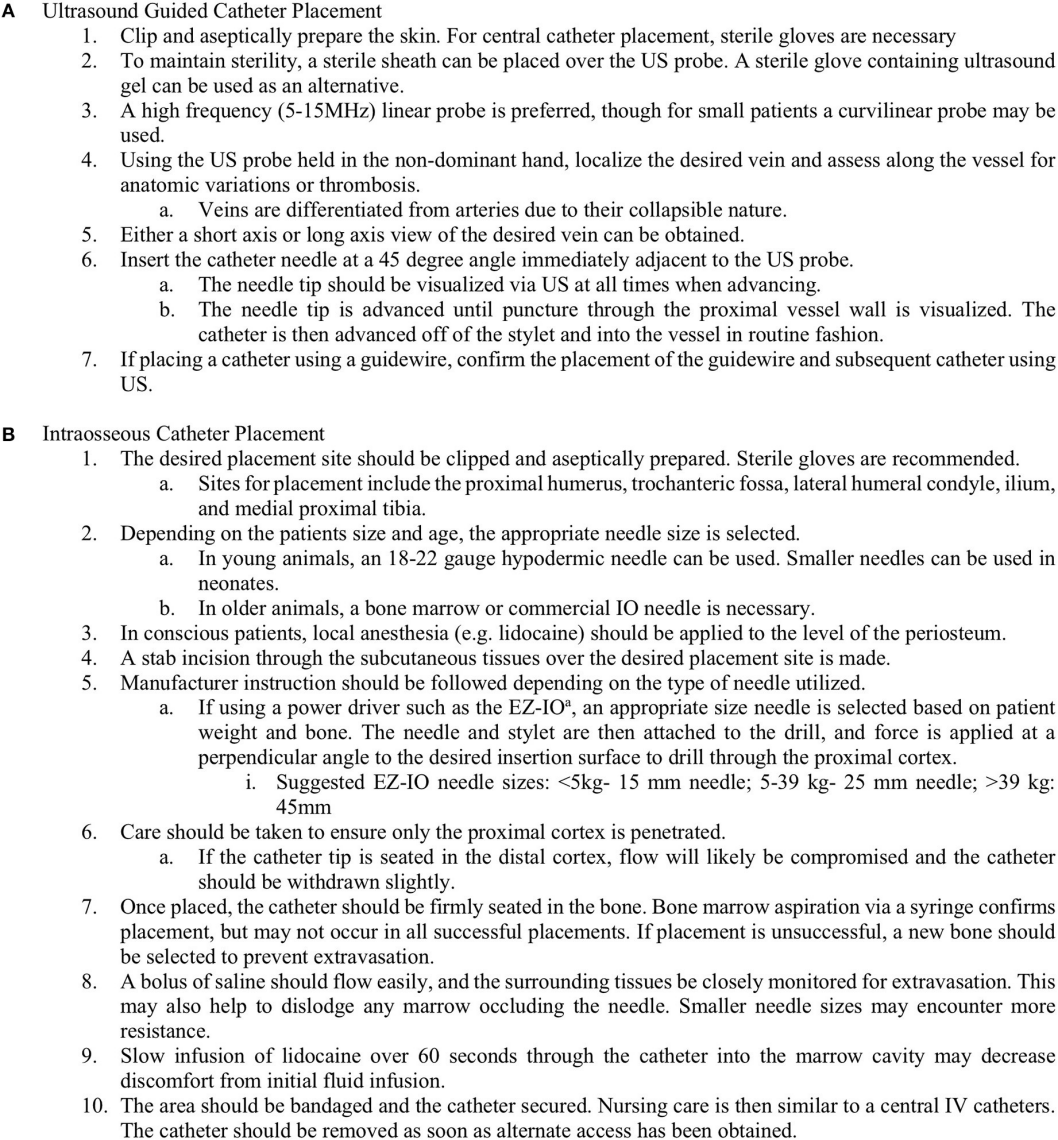
Procedure for ultrasound-guided catheter placement **(A)** and intraosseous catheter placement **(B)** in dogs and cats in order to obtain emergency vascular access.

Ultrasound (US) guidance has been incorporated in both peripheral and central venous access procedures. High-quality US is becoming increasingly available in veterinary medicine. Daily point-of-care use in the emergency room (ER) and intensive care unit (ICU) is increasing, and the cost of this equipment is decreasing. Ultrasonography provides practitioners with the ability to visualize the vessel for catheterization. This can be particularly helpful when palpation or visualization of vascular landmarks is difficult. It can also allow for identification of any abnormal surrounding anatomy. High-frequency linear probes (>4 MHz) are most commonly used for this purpose, though convex probes have been used in small patients. This technique has been well-described in people and is described here in Figure 1A (37–39). To maintain sterility, the probe is placed within a sterile sheath or sterile glove containing US gel (40–42). Application of alcohol to the intended site can be used to improve visualization. Vessels may be differentiated using Doppler imaging to detect pulsatile flow in the vessel (43). Arteries can be differentiated from veins due to their non-collapsible nature (37). After clipping and aseptic preparation of the desired site, the probe is generally oriented either transversely (short axis) or longitudinally (long axis) to the vessel, and the needle is advanced through the skin from several centimeters away from the probe (21–23). The use of a real-time approach to advance the needle is preferred over using the US to locate a landmark prior to non-US-guided needle insertion. Evidence regarding superiority of one orientation of the probe in the human literature has been mixed (42, 44–48). Meta-analyses of probe orientation for multiple US-guided vascular access sites failed to find any significant difference for the first-pass success rate, mean time to success, mean attempts to success, and incidence of hematoma formation (49). Each view may have benefits, and developing familiarity with both is useful. The transverse view allowed for superior identification of other vessels but shows only a cross section of the desired vessel, making puncture more challenging. In contrast, a longitudinal view of the vessel allows for monitoring of the needle as it is advanced, though precise alignment of the plane of the probe and the course of the needle is needed, and visualization of other vessels or nerves may be lost. An oblique technique has also been described, which may combine the advantages of each view, but requires greater familiarity with US (42).

In human medicine, meta-analysis of US-guided catheter placement showed improved success of catheter placement, increased speed of placement, and decreased rate of complications as compared with blind or landmark-based placement, including in emergency patients (27, 29). The best evidence exists in aiding central venous catheterization, though benefit has also been shown with peripheral access (22, 41, 50). US guidance is therefore increasingly recommended as the standard of care (24, 41–43, 51, 52). In veterinary medicine, US guidance is still being investigated. A study in healthy anesthetized canines did not find an improvement in the time to vascular access (45 s for US guidance vs. 7 s for landmark placed) or success (97 vs. 95%) of central jugular catheter placement compared with a landmark-based technique (40). More applicably to critically ill patients, an experimental cardiopulmonary resuscitation (CPR) model in dogs demonstrated feasibility of ultrasound-guided jugular access with an average time to vascular access of 2–4 min (43). Further studies are needed to evaluate the feasibility and benefits of such a technique in critically ill dogs and cats.

## Intraosseous Access

Because of the non-compressible nature of bone, intraosseous (IO) access offers a consistent route of access to the systemic circulation in the face of hypotension and hemodynamic collapse. While initially implemented largely in human pediatrics, use was expanded to adult patients (53). It is increasingly being advocated as a first-line option in both the pre-hospital and ER settings when rapid peripheral IV access is unsuccessful, including during CPR (53). Depending on patient size, access can be obtained using the stylet of an over-the-needle IV catheter or hypodermic needle (both reserved for young patients), using a manually placed bone marrow needle (e.g., Jamshidi), or using a purpose built IO catheter, often inserted *via* a bone injection gun or proprietary power driver (24, 27, 54). The latter modality is a semiautomated device that has been shown to improve speed of placement (54–57). Limitations of placement include need for specialized equipment (except in case of using a hypodermic needle), which may restrict availability. Flow rate is also limited by needle diameter and the bone selected (58). Contraindications include osteomyelitis, regional pyoderma, preexisting fracture, and orthopedic hardware in the location of interest (53, 54). Possible complications reported in humans include osteomyelitis, fat embolism, fluid extravasation, nerve injury, compartment syndrome, and bone fractures. The overall rate of complications in humans has been reported at <1% (53, 59, 60), with the most common being extravasation of fluid, which can result in local tissue damage depending on the extravasated substance and potentially compartment syndrome (53, 61). Compartment syndrome has not been reported in dogs or cats with IO catheterization. However, careful monitoring for extravasation is imperative. Overall complication rates have not been documented in dogs and cats (54).

**Figure 2:**
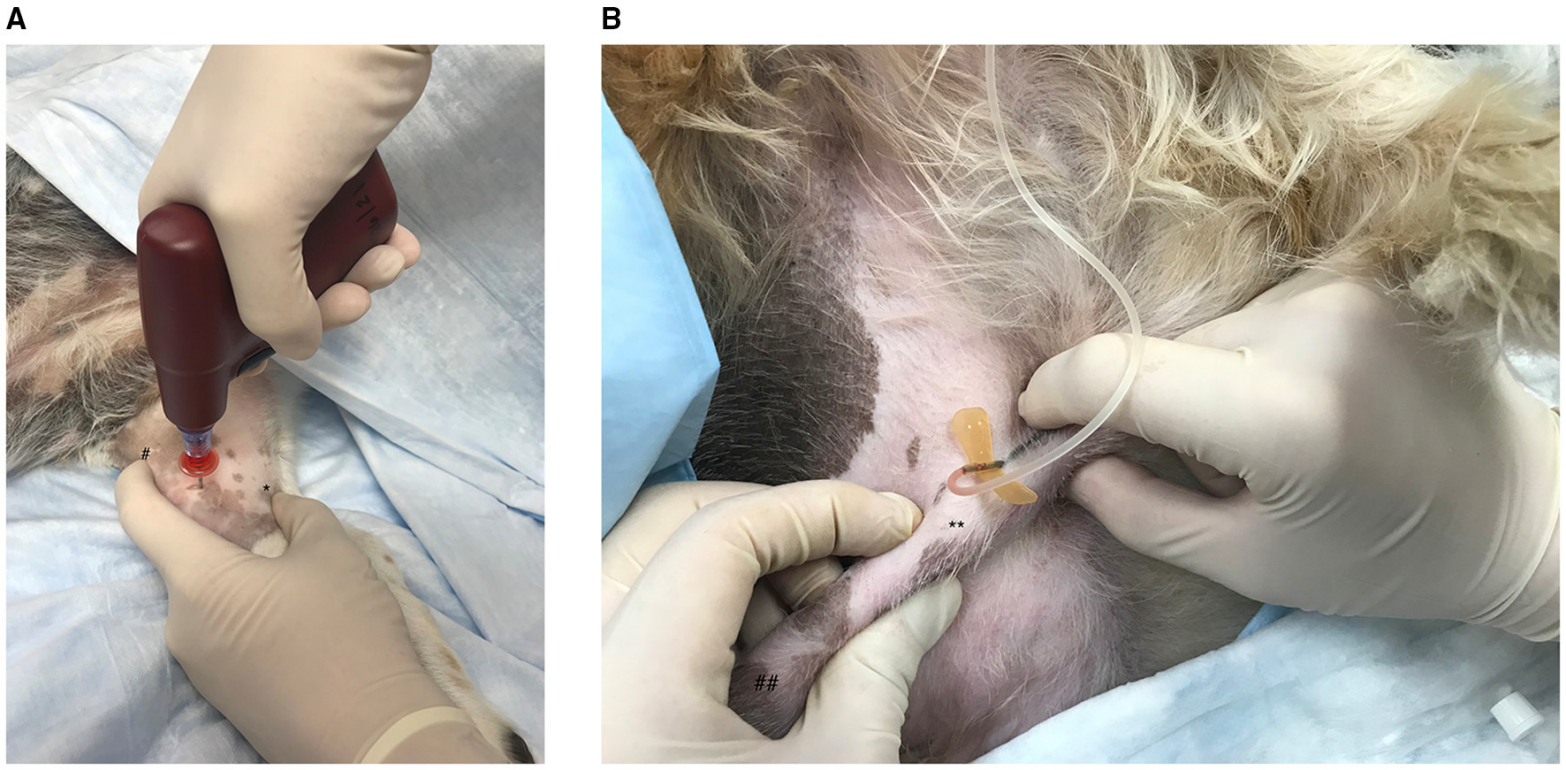
**(A)** Emergency vascular access *via* intraosseous catheterization in the canine medial tibia using the EZ-IO G3 Power Driver (Vidacare Corp., Shavano Park, TX, USA). After aseptic preparation of the skin, a scalpel blade is used to make a small skin incision over the desired area. In a right-handed operator, the left index figure is used to palpate the patellar tendon (#) to identify and avoid the stifle joint then placed directly distal to the joint on the cranial aspect of the tibia. The left thumb is then used to identify and secure the caudal aspect of the tibia (*). The remaining fingers of the left hand are used to stabilize the distal tibia. The G3 Power Driver is held in the right hand and positioned perpendicular to the tibia. The catheter is then advanced through the bone until a drop in resistance indicates the catheter has entered the medullary cavity. The catheter should be well-seated in the bone. Bone marrow aspiration *via* a syringe confirms placement. A bolus of saline should flow easily, and the surrounding tissues should be closely monitored for extravasation. **(B)** Placement of a catheter in the corpus cavernosum of a male canine. With the patient in lateral recumbency, the penis is isolated within the prepuce at the level of the caudal os penis (**). The pars longa glandis is on the left (##). The needle is then inserted into the corpus cavernosum *via* the lateral aspect of the penis at an approximately 45° angle, directed caudally. Aspiration of blood and easy flow of saline through the catheter confirm placement.

In dogs, successful placement has been described in the proximal humerus, lateral humeral condyle, trochanteric fossa, wing of the ilium, and medial proximal tibia (27, 54, 58). A canine cadaveric study found the proximal humerus and distal femur to have the best combination of high flow rates and ease of access (58). IO access has also been investigated in cats at the medial tibia and proximal humerus (56). Placement in the pelvic limbs may be desirable in a CPR setting, where chest compression and airway manipulation complicate attempts to access the thoracic limbs, although the greater distance between this site and the heart is a consideration given poor circulation (54). This technique is described in detail in Figures 1B, 2. Time of placement in a human cadaver model was faster than peripheral venous cutdown (3.9 vs. 7.6 min) (31). The technique appears to have a rapid learning curve, with reported success rates of 87.5% in a canine cadaver study (62). In that study, time to IO placement was also faster than jugular cutdown (0.9 vs. 3.6 min) (62). Infusion of medications into the bone marrow can be painful, as is removal of the catheter. Administration of hypertonic solutions *via* this route is controversial due to the potential for marrow and muscle necroses (63). However, most CPR medications, isotonic fluids, and blood products can be safely administered (27, 53, 54, 56, 63, 64). These are rapidly absorbed in the central circulation, with comparable pharmacokinetics to IV administration (65). While IO catheters may remain in place for up to 72 h, they should be removed as soon as another vascular access route has been established (54, 66, 67).

Of additional interest, there has been recent research regarding the ability to derive point-of-care clinicopathologic information from marrow aspirated from the IO catheter at the time of placement. Studies in hemodynamically stable pigs, children, and adults have shown varying agreements between IO and venous samples for different analytes (57, 65, 68, 69). Only a few human studies focusing on hemodynamically unstable patients have been published to date. One group performed minimum database analysis measurements in hemodynamically unstable patients and found a clinically acceptable agreement for pH, bicarbonate, base excess, and sodium and a moderate correlation for lactate and glucose, while pCO_2_, pO_2_, and potassium concentration did not show good agreement when IO and venous samples were compared (70). In another study, agreement declined after 15 min of CPR, particularly for acid base status (69). A recent veterinary study performed in healthy dogs anesthetized for orthopedic surgery demonstrated good agreement between IO aspirates and venous samples to assess minimum database variables [blood gas tensions, electrolytes, lactate, blood urea nitrogen (BUN), glucose, and packed cell volume/total protein (PCV/TP)], but not potassium or hematocrit (71). No veterinary paper has investigated the utility of IO samples in hemodynamically unstable dogs or cats. This would benefit from further study in critically ill patients.

## Umbilical Vein

In neonates, the umbilical vein (UV) provides an alternative route of access. The viability of the vessel depends on whether it has been previously ligated and is likely to be most accessible in the first day of life. This technique utilizes readily available material but can be more time-consuming and more difficult to perform than some alternatives if not routinely practiced and in the tight space constraints of a resuscitation setting (72). Contraindications include abnormal umbilical anatomy, omphalitis, and septic peritonitis (73, 74). Access is gained by first encircling the base of the umbilicus with umbilical tape and tightening to prevent hemorrhage. With the use of sterile technique, the umbilicus is then incised to expose the large, thin-walled vein, as well as the two smaller thick-walled umbilical arteries. A catheter is then advanced into the vein (73–75). It may be most practical to use the UV for single injections, which is readily achieved using a 25-G needle. Care must be taken not to advance the catheter more than a few centimeters beyond the level at which a flash of blood is seen, as there is the potential for the catheter to lodge within the portal system if advanced too far into the central circulation without checking placement using a radiograph (72, 74, 76). US-based placement confirmation has also been described in humans (77). Other complications include extravasation, thromboembolism, perforation of the peritoneum, and ischemia (72, 78).

In human neonates, IO is faster than UV catheterization in simulated resuscitation models, in both experienced and inexperienced hands (79–81). The average time required for IO catheter placement was ~1–2 min faster than for UV catheter placement in simulated neonatal resuscitation (72, 79). Another study showed similar results and greater subjective ease of placement of the IO catheter in novice operators (80). Nevertheless, it is an important tool for vascular access in neonates when other routes are not available. Human studies comparing umbilical access with other routes in a clinical setting appear to be lacking, as are veterinary-specific studies.

## Conclusion

In conclusion, multiple options exist for patients with DVA. In particular, US-guided vascular access and IO catheters have become more readily available to practitioners, and both offer potential advantages. Further research is needed to verify their utility in unstable veterinary patients. Additionally, further research into DVA in veterinary patients could help to more rapidly identify patients that would benefit from these techniques.

## Author Contributions

JL, L-VG, GB, and CS contributed to the conception and design of the manuscript. JL wrote the first draft of the manuscript. All authors contributed to manuscript revision and read and approved the submitted version.

## Funding

The funding for publication costs was provided by the University of Tennessee Veterinary Teaching Hospital Small Animal Clinical Sciences Department.

## Conflict of Interest

The authors declare that the research was conducted in the absence of any commercial or financial relationships that could be construed as a potential conflict of interest.

## Publisher's Note

All claims expressed in this article are solely those of the authors and do not necessarily represent those of their affiliated organizations, or those of the publisher, the editors and the reviewers. Any product that may be evaluated in this article, or claim that may be made by its manufacturer, is not guaranteed or endorsed by the publisher.

## Abbreviations

BUN: blood urea nitrogen
DVA: difficult vascular access
IO: intraosseous
IV: intravenous
PCV: packed cell volume
TP: total protein
US: ultrasound
UV: umbilical vein.
